# Lynch syndrome cancer vaccines: A roadmap for the development of precision immunoprevention strategies

**DOI:** 10.3389/fonc.2023.1147590

**Published:** 2023-03-22

**Authors:** Shizuko Sei, Aysel Ahadova, Derin B. Keskin, Lena Bohaumilitzky, Johannes Gebert, Magnus von Knebel Doeberitz, Steven M. Lipkin, Matthias Kloor

**Affiliations:** ^1^ Division of Cancer Prevention, National Cancer Institute, National Institutes of Health, Rockville, MD, United States; ^2^ Department of Applied Tumor Biology, Institute of Pathology, Heidelberg University Hospital, Heidelberg, Germany; ^3^ Clinical Cooperation Unit Applied Tumor Biology, German Cancer Research Center Deutsches Krebsforschungszentrum (DKFZ), Heidelberg, Germany; ^4^ Translational Immunogenomics Laboratory, Dana-Farber Cancer Institute, Boston, MA, United States; ^5^ Department of Medical Oncology, Dana-Farber Cancer Institute, Boston, MA, United States; ^6^ Broad Institute of The Massachusetts Institute of Technology (MIT) and Harvard, Cambridge, MA, United States; ^7^ Department of Computer Science, Metropolitan College, Boston University, Boston, MA, United States; ^8^ Harvard Medical School, Boston, MA, United States; ^9^ Section for Bioinformatics, Department of Health Technology, Technical University of Denmark, Lyngby, Denmark; ^10^ Joan and Sanford I. Weill Department of Medicine, Weill Cornell Medical College, New York, NY, United States

**Keywords:** lynch syndrome, DNA mismatch repair deficiency, microsatellite instability, frameshift mutations, tumor neoantigens, cancer vaccines, immunoprevention, precision cancer prevention

## Abstract

Hereditary cancer syndromes (HCS) account for 5~10% of all cancer diagnosis. Lynch syndrome (LS) is one of the most common HCS, caused by germline mutations in the DNA mismatch repair (MMR) genes. Even with prospective cancer surveillance, LS is associated with up to 50% lifetime risk of colorectal, endometrial, and other cancers. While significant progress has been made in the timely identification of germline pathogenic variant carriers and monitoring and early detection of precancerous lesions, cancer-risk reduction strategies are still centered around endoscopic or surgical removal of neoplastic lesions and susceptible organs. Safe and effective cancer prevention strategies are critically needed to improve the life quality and longevity of LS and other HCS carriers. The era of precision oncology driven by recent technological advances in tumor molecular profiling and a better understanding of genetic risk factors has transformed cancer prevention approaches for at-risk individuals, including LS carriers. MMR deficiency leads to the accumulation of insertion and deletion mutations in microsatellites (MS), which are particularly prone to DNA polymerase slippage during DNA replication. Mutations in coding MS give rise to frameshift peptides (FSP) that are recognized by the immune system as neoantigens. Due to clonal evolution, LS tumors share a set of recurrent and predictable FSP neoantigens in the same and in different LS patients. Cancer vaccines composed of commonly recurring FSP neoantigens selected through prediction algorithms have been clinically evaluated in LS carriers and proven safe and immunogenic. Preclinically analogous FSP vaccines have been shown to elicit FSP-directed immune responses and exert tumor-preventive efficacy in murine models of LS. While the immunopreventive efficacy of “off-the-shelf” vaccines consisting of commonly recurring FSP antigens is currently investigated in LS clinical trials, the feasibility and utility of personalized FSP vaccines with individual HLA-restricted epitopes are being explored for more precise targeting. Here, we discuss recent advances in precision cancer immunoprevention approaches, emerging enabling technologies, research gaps, and implementation barriers toward clinical translation of risk-tailored prevention strategies for LS carriers. We will also discuss the feasibility and practicality of next-generation cancer vaccines that are based on personalized immunogenic epitopes for precision cancer immunoprevention.

## Introduction

Cancer prevention strategies are generally centered around the reduction of cancer risks. Modifiable cancer risk factors include tobacco use, alcohol consumption, obesity, diabetes, and infection with oncogenic viruses, such as human papillomaviruses (HPV) and hepatitis B virus (HBV). Lifestyle changes and receiving prophylactic vaccines against HPV and HBV can significantly reduce these risks (1). In contrast, genetic predisposition to cancer is not modifiable. Individuals with hereditary cancer syndromes (HCS) account for 5 to 10% of all cancer cases (2). They are clinically identifiable by genetic testing (3–5), are well characterized with predictable ages of disease onset, organ involvement, and molecular pathophysiology, and can be closely monitored for early cancer detection and diagnosis based on HCS guidelines (6). Cancer risk mitigation strategies for HCS carriers, therefore, include primary prevention of cancer as well as detection and elimination of cancer precursors and early-stage (*in situ*) cancers before they progress to invasive cancers, the approach referred to as cancer interception (7). While much progress has been made in the development of new or improved methods of detecting cancer early (8), with the exception of aspirin for Lynch syndrome (LS) (9) there are currently no effective cancer preventive or interceptive approaches available to them other than surgical (endoscopic or surgical) removals. Most of the conventional anticancer therapeutic agents are too toxic for cancer interception.

Vaccine-preventable infectious diseases (10) are a good example of illnesses that can be safely prevented if vaccines are used as recommended. The unprecedented speed of successful development and deployment of COVID-19 mRNA vaccines in 2020 ~ 2021 was a tremendous scientific achievement that had culminated from years of research in relevant scientific disciplines, including coronavirus virology, vaccinology, and innovative mRNA vaccine technology, and a strong public-private partnership (11, 12). Prerequisites for successful vaccine development generally include identification and characterization of causative agents, understanding of disease pathogenesis and pathophysiology, and availability of suitable preclinical tools and animal models, which recapitulate human disease conditions and host immune responses and therefore can provide a proof of principle for *in vivo* vaccine efficacy. Compared to prophylactic vaccines against infectious pathogens that have generally fulfilled these prerequisites throughout the history of vaccine development (13), the development of cancer vaccines in general has been met with more challenges (14).

The effect of cancer vaccine was first evaluated in cancer patients in 1959 (15) shortly after Burnet postulated the concept of cancer immunosurveillance (16). While important advancements were made in cancer immunology and vaccinology over the last several decades, the majority of cancer vaccine research has focused on eliciting effective antitumor immunity to treat advanced cancer (17). Various therapeutic cancer vaccines were extensively evaluated in patients with advanced cancer with little success, likely due to the immunosuppressive factors locally in the tumor microenvironment (TME) and systemically (18). The concept of cancer vaccines for immunoprevention started gaining more traction in the last 20 years owing to the pioneering work of Finn, Disis, and others against non-viral cancers (14, 19–25). Target antigens selected for cancer preventive vaccines have predominantly been tumor-associated or tumor-specific antigens that are overexpressed or specifically expressed in cancer precursors and cancer cells and proven immunogenic across different HLA types (26, 27). Cancer vaccines with such commonly expressed (shared) tumor antigens can be more easily studied for efficacy in a well-defined high-risk cohort and thus can be streamlined for further development.

More recently, successful immunotherapy outcomes with immune checkpoint inhibitors (ICI) for various cancers have clearly shown that the immune system can mount strong antitumor immune responses leading to complete remission in some cases if systemic and local immunosuppression in the TME is effectively blocked (28, 29). Interestingly, antitumor immune responses unleashed by immune checkpoint blockade have been shown to target a large repertoire of tumor antigens that are unique to individual patients (i.e., personalized antigens) (30–32). It is conceivable that more robust and durable antitumor immunity can be elicited by cancer vaccines in the prevention or interception setting, wherein local immunity in the TME is less compromised and there is still low clonal heterogeneity of tumor antigens (22, 33, 34). Naturally, questions arise as to whether immunopreventive cancer vaccines can be developed based on personalized tumor antigens and whether such personalized vaccines are more efficacious and desirable than cancer vaccines that target shared/common tumor antigens. This review will discuss the development of LS vaccines as a model strategy for preventing cancer in HCS cohorts, emerging enabling technologies, research gaps and implementation barriers for cancer immunoprevention, and research trajectory towards next-generation precision cancer vaccines for immunoprevention.

## Precision cancer prevention

Apart from the modifiable risk reduction strategies discussed earlier, cancer prevention for high-risk cohorts can be improved by determination of risks based on oncogenic mechanisms inherent to a specific cohort, closer monitoring of affected individuals for early cancer detection, and timely and effective interventions developed specifically for each high-risk group. These risk-tailored cancer prevention strategies are interchangeably referred to as personalized or precision cancer prevention. For the purpose of this review, which is focused on cancer prevention strategies for HCS cohorts, in particular LS, we define precision cancer prevention as risk-tailored cancer prevention strategies informed by underlying oncogenic mechanisms responsible for the development and progression of cancer and molecular alterations targetable for cancer prevention and interception in high-risk populations (35). We will use the term “personalized” when we refer to tumor antigens unique to each individual as opposed to shared or commonly expressed antigens (36).

The concept of precision oncology was originally introduced as cancer genomics-informed “personalized or precision” cancer medicine to facilitate the decision on treatment choices for individual cancer patients (37). The common denominators of precision cancer prevention and precision cancer medicine strategies are the involvement of molecular and immune mechanisms of oncogenesis in the decision-making process for interventions rationally and uniquely developed for individuals. Neoantigens are newly acquired and expressed “non-self” antigens arising from gene mutations, exogenous genes (e.g., viral proteins), or alternative antigen processing. The host immune system recognizes these neo-peptides presented with MHC molecules on the cell surface as non-self, mount immune responses against them, and eliminate the neoantigen-expressing aberrant cells from the body (38). During tumor development and progression, tumors accumulate numerous gene mutations, which, if translated, give rise to neoantigens (39, 40). These neoantigens expressed in cancers can be targeted by the host immune system for surveillance and elimination. Although it’s been long postulated that tumor neoantigens would serve as promising cancer vaccine antigens, the discovery and neoepitope selection was a major hurdle until recently. The advent of next-generation sequencing technologies and rapid development of powerful computational analytical tools, which enable comprehensive “mutanome” analysis of individual tumors and the identification of personalized immunogenic neoepitopes, has led to seminal neoantigen cancer vaccine studies in melanoma patients either as peptide-based (41) or mRNA-based vaccines (42). Both studies demonstrated immunized patients mounted robust T cell responses to unique neoantigens, associated with prolonged and objective responses in some cases. The long-term outcome study of patients who received personal neoantigen vaccines demonstrated the clinical benefit at a median follow-up of 4 years post-vaccination and long-term persistence of memory T cells specific to personal neoantigens as well as the evidence of epitope spreading (43).

While there is mounting evidence to suggest that immune responses directed against personal neoantigens can block or control cancer growth and clinically benefit vaccinated patients (30–32, 41–43), the approach cannot be generally translated into the prevention setting unless tumor neoantigens could be “predicted” in individuals who have yet to develop cancers. The first breakthrough observation was made by Kloor, von Knebel Doeberitz, and colleagues, who demonstrated that insertion/deletion frameshift (FS) mutations could be predicted based on the known genetic sequences of coding microsatellites (cMS) and that the specific mutation frequencies could be evaluated in LS/mismatch repair deficiency (MMRd)-associated tumors (44, 45). They discovered that colonic adenomas from LS carriers harbored MMRd-driven FS mutations in the cMS regions at high frequencies for certain genes at levels similar to those found in colorectal carcinomas (46). They further demonstrated frameshift peptides (FSP)-specific T cell responses could be observed not only in LS patients with colorectal cancer (CRC), but also in cancer-free asymptomatic LS carriers (47). These findings clearly demonstrated that LS carriers harbored FSP neoantigens before the onset of overt CRC tumorigenesis and the host immune system was capable of mounting anti-FSP immunity, which may play a role in keeping tumor growth in check. Using commonly recurring (broadly shared) FSP (rFSP) neoantigens as vaccine antigens, they subsequently proposed an rFSP neoantigen-based cancer vaccine for MMRd cancers and successfully demonstrated the safety and immunogenicity in Phase I/IIa clinical trial (48), providing the proof of principle of rFSP neoantigen-based cancer vaccine strategies for cancer prevention and interception in LS carriers.

Tumor-specific neoantigens can be vastly heterogenous and immune responses are restricted by MHC molecules (38). Their expression levels also vary among different neoantigens. Because of the intra- and inter-individual heterogeneity of tumor-specific neoantigens and the diversity of immune responses that are determined by HLA alleles, it is extremely challenging, if not impossible, to develop broadly applicable cancer vaccines targeting shared neoantigens for different HCS carriers, with LS being an exception as discussed earlier. In this regard, the development of personalized cancer preventive vaccines may be more straightforward. Similar to the approach used for the development of precision cancer therapeutic vaccines (41, 42), personalized immunogenic epitopes for preventive vaccines can be identified from molecular and immuno-neoepitope analysis of precancerous lesions. The question, however, is whether there is an advantage to personalize FSP-based cancer preventive vaccines for individual LS carriers when shared FSP antigens can be readily identified. Considering the amount of time and resources required to generate such personalized FSP vaccines for nearly one million LS carriers in the US alone, the concept of personalized immunopreventive cancer vaccines is prohibitively impractical for the LS cohort at this time. At the same time, the debate on the use of personalized neoantigen based vaccines for cancer prevention should also involve the lifetime disease severity and progression trajectory. For example, consider children with constitutional mismatch repair deficiency (CMMRD) syndrome, one of the most aggressive forms of childhood cancer predisposition syndromes, resulting from biallelic deleterious germline mutations in the MMR genes (49). As in LS, DNA MMR deficiency can trigger FS mutations in the cMS, giving rise to FSP neoantigens in these children (50). Children with CMMRD develop brain tumors, hematological malignancies (in particular, non-Hodgkin lymphomas of T-cell lineage, T cell ALL and AML), gastrointestinal and other LS-associated cancers, sarcomas (e.g., osteosarcoma and rhabdomyosarcoma), and other childhood cancers (e.g., neuroblastoma and Wilms tumor) (49, 51). Cancers arising in children with CMMRD have the highest mutational and MS insertion-deletion (MS-indel) burden, are resistant to chemo-radiation interventions, and considered lethal (52). Children with CMMRD therefore may clinically benefit from receiving personalized cancer preventive vaccines. If we aim to develop and deploy risk-tailored and risk-weighted precision cancer prevention strategies, the critical first step is to identify the genetic predisposition carriers and investigate the pathophysiology of oncogenesis in each HCS population.

## Advances in genetic predisposition screening technologies

Of the ~140,000 new diagnoses of CRC each year in the United States, ~25% to 30% of patients diagnosed have a first or second degree relative (parents, siblings, children, uncles, aunts and first cousins) with CRC (53). The most common inherited CRC syndromes is LS, which is diagnosed by germline autosomal dominant mutations of DNA MMR genes, *MLH1, MSH2, MSH6 and PMS2*, or structural variations in *EPCAM* that drive MSH2 epigenetic inactivation (54, 55). LS is estimated to occur in approximately 1:280 individuals (56). A related syndrome is CMMRD discussed above, a much less frequent pediatric autosomal recessive disease where children inherit bi-allelic MLH1, MSH2, MSH6 or PMS2 mutations that drive aggressive cancer predisposition and are affected with cancers as often as every 2-3 years in early life and commonly perish from brain, GI, and hematopoietic malignancies (50). Additionally, autosomal recessive mutations of MMR genes, MSH3 or MLH3, cause colorectal polyposis and CRC, which is a separate syndrome characterized by distinct patient phenotype and familial inheritance pattern (57–60). This review section focuses on LS.

Historically, LS was referred to as hereditary non-polyposis colorectal cancer (HNPCC). However, LS is now preferred to highlight that that these patients and their families have higher rates of multiple other cancers, most notably endometrial and gastric cancers, but also including ovarian, pancreatic-biliary, urinary tract (kidney, renal pelvis, ureter, bladder, and prostate), small intestinal, brain cancers and sebaceous neoplasms of the skin, among others (61).

Overall, there are two primary strategies for diagnosis of LS. Historically, family history followed by germline DNA mutation testing performed on patients with personal or family history of cancer suspicious for LS was the primary approach. This approach uses different clinical criteria, most notably the Amsterdam or Bethesda criteria (53), both of which focus on age on onset for CRC, and family cancer history of first-, second- and third-degree relatives for LS associated cancers. However, family history taking, while virtually costless and in principle universally implemented, was found to significantly underdiagnose LS (62–65). In part, at least in the United States, this is driven by growing provider economic and corporate medical pressures on primary care physicians to rapidly provide comprehensive medical care for ever growing panels of patients (which can average 1 patient every 15 minutes in some clinics anecdotally) and primarily focus on symptomatic crises rather than less urgent preventative medical care that shortchange history and disease interception strategies. Additionally, there are non-trivial rates of non-paternity, which range from ~0.4% to as high as 30% in different populations but roughly averaging to ~1% across different societies (66, 67), further confounding family history taking. Most importantly, population screening studies of CRC and endometrial cancer (68, 69), primarily by immunohistochemistry (IHC) testing, provided direct data revealing that many LS patients had limited family history and/or later onset cancers. In addition to the practical health services implementation issues with family history taking discussed above, this is driven by the fact that family history does not capture patients who have *de novo* LS mutations (aka, a new mutation not inherited from either biological parent) and because many LS patients carry MSH6 or PMS2 mutations, which confer lower overall lifetime cancer risk than MLH1 or MSH2 mutation carriers (54, 56).

Underdiagnosis of LS is not unique to the United States. It also remains a challenging issue in Europe (70, 71). A Swedish study has demonstrated that one third of LS patients referred for genetic testing already had cancer, indicating that these individuals’ genetic risk was unknown until they developed LS-associated cancers (72). This proportion of individuals diagnosed with LS due to cancer diagnosis has not changed over the decades (72). A survey across 14 Western European countries showed that the quality of family history taking was thought to be generally poor and there were virtually no specific campaigns or strategies in place to increase the public awareness of hereditary cancers except in one country (Germany) (73). Another study has also reported that family history has been poorly documented even in the electronic health record (74) contributing to the deficiency in family history taking approach. Thus, while personal and family history taking approach has been the cornerstone of LS diagnosis, it significantly undercounts its prevalence.

The second primary approach to LS diagnosis starts not with clinical criteria per se, but molecular screening of CRC, endometrial, pancreatic and other tumor specimens for evidence of MMRd from patients who are diagnosed with carcinomas. This can be performed as polymerase chain reaction (PCR)-based microsatellite instability (MSI) testing of newly or previously archived tumors after diagnosis, immunohistochemistry (IHC) for MMR genes, or direct tumor gene panel sequencing and mutation burden analysis to identify patients who then have germline testing for MMR gene mutations (which is sometimes but not always done simultaneously with tumor sequencing) (54). Importantly, these molecularly initiated approaches also identify patients who have sporadic (aka non-LS) MMRd tumors, which arise *via* somatic (non-CMMRD congenital) bi-allelic mutation and/or epigenetic inactivation of MMR genes, primarily MLH1 (54).

Cascade testing involves targeted mutation analysis testing of blood relatives from patients who are affected by a genetic disease (4, 75–77). Recent clinical trial and cost effectiveness research studies have provided evidence that primary molecular screening augmented by follow-up targeted cascade testing of family members of affected LS (and other genetic disease) probands is a cost effective public health strategy to diagnose a high percentage of LS mutation carriers that is predicted to identify almost all affected individuals after approximately a decade of implementation (4, 75–77).

Analysis of circulating cell-free tumor DNA (cfDNA) using liquid biopsy has enabled non-invasive detection of tumor mutations. Currently, liquid biopsy is an established technology to replace and/or augment tumor biopsy sequencing, including the detection of minimal residual disease (MRD) after surgery or chemotherapy. Liquid biopsy can be used for detection of LS germline mutations (78) and for detecting MMRd tumors, which carry very elevated tumor mutation burdens. Currently, the highest sensitivity for liquid biopsy detection of MMRd tumor DNA is shown by low pass whole genome sequencing of cfDNA and mutation signature analysis (79), which draws on sequence data from low pass coverage sampling of the ~23,000,000 microsatellites encoded in the human genome (50). However further studies, and perhaps additional augmentation by orthogonal technologies including protein and microbial analyte data streams, are needed before this population surveillance strategy becomes useful for LS diagnosis, screening, and surveillance.

Because of the high tumor mutation burden, it has been long been appreciated that MMRd tumors have elevated numbers of tumor infiltrating lymphocytes (TILs) and other enriched histopathology features of the tumor microenvironment (54). Recently, there has been evidence that machine learning of histopathology images of tumors can detect “MMRdness (80)”. However, although innovative and expected to improve in the future with advances in computational analysis, the sensitivity and specificity of ML histopathology detection of MMRd status may currently be problematic and not just yet ready for clinical application (81).

In summary, identification of LS is important because it is a clinically actionable diagnosis driving increased tumor surveillance, chemoprevention [primarily aspirin, discussed at length elsewhere (9)] and post-tumor therapy choice [immune checkpoint inhibition, discussed at length elsewhere (82)] in a high-risk cancer population. Screening and diagnosis of LS mutation carriers is an evolving paradigm that begins with personal and family history as its cornerstone, but requires additional molecular, computational and health services analyses to reach its potential for improving survival for affected patients. This point is particularly true regarding underrepresented and underserved minority populations, which often have lower rates of information collection or access from detailed family history taking, in addition to less access to preventative medical care and cost-intensive medical technologies (62–65).

## Lynch syndrome vaccines for immunoprevention

LS is an ideal model disease for testing the potential of cancer immunopreventive approaches due to the pathogenesis of LS-associated tumors. The penetrance of the disease varies widely depending on the MMR gene affected in the germline, with *MLH1* and *MSH2* genes being associated with relatively high cancer risk (about 50%: Prospective Lynch Syndrome Database or PLSD) (83) and found in about 90% of LS-associated tumors. In addition to the affected MMR gene, further factors are suspected to influence individual cancer risk in LS carriers, as also within-gene and even within-family differences in cancer risk have been observed. These factors include other genetic constellations (polygenic risk score) and environmental influences. More recently, the possible influence of the immunological factors has also been gaining substantial attention, which is related to the growing knowledge about a close interplay between arising MMR-deficient tumor cell clones and the immune microenvironment.

The driving force of carcinogenesis in LS is MMRd, leading to the inability of affected cells to repair base mismatches occurring during DNA replication. When unrepaired, such errors cause indel mutations, typically indels of one or two nucleotides at short repetitive DNA stretches (microsatellites). The molecular phenotype of MMR-deficient cells is characterized by the accumulation of insertion/deletion mutations at microsatellites and called MSI as discussed earlier. Microsatellite regions are distributed over the entire human genome. Because approximately 99% of the genome has no protein-coding function, most microsatellite mutations do not have an immediate effect on a cell’s functional phenotype. However, mutations at microsatellites in protein-coding genomic regions (coding microsatellites, cMS), which are mostly mononucleotide repeats, can have drastic consequences on the function of the encoded protein and its immunological properties. Due to an insertion or a deletion of one or two nucleotides, the entire subsequent reading frame is shifted (frameshift mutations). This can lead to premature stop codons and translation of truncated, non-functional proteins. cMS mutations affecting tumor suppressor genes can drive tumorigenesis in the mutated cell. Simultaneously, the newly translated FSP sequence often contains numerous epitopes, which are foreign to the host’s immune system, rendering the affected cells highly immunogenic.

**Figure 1:**
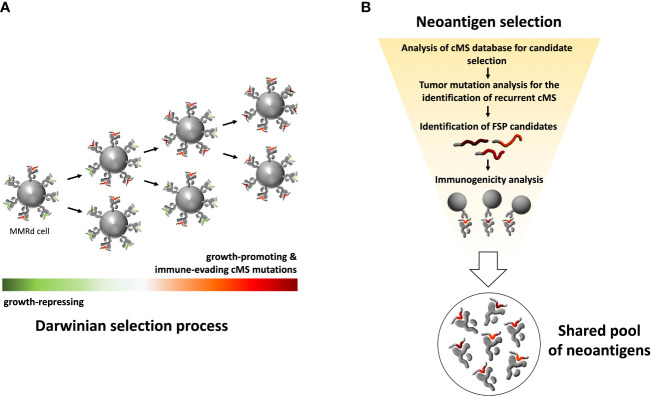
Schematic illustration of MMRd tumor clone selection process and workflow for selecting a pool of shared immunogenic neoantigens. **(A)** Schematic illustration of Darwinian selection process underlying MMRd cancer evolution. The random accumulation of indel mutations in cMS, caused by impairment of the MMR system, is followed by a non-random persistence of mutations. Cell clones carrying mutations that promote tumor outgrowth or provide other survival advantages, such as immune evasion, are positively selected. This evolutionary selection of mutations leads to recurrent cMS mutation patterns in MMRd cancers and thus a predictable pool of FSPs. **(B)** Strategy for the selection of shared, immunogenic FSPs for immunoprevention. Screening of a genome-wide cMS database forms the basis for the identification of recurrent cMS mutations shared by MMRd cancers. Accounting for mutation frequency in MMRd cancers, immunogenicity prediction in silico and immunogenicity testing *in vitro*, a pool of candidate neoantigens can be selected for FSP vaccines.

Thus, the carcinogenic process of LS cancers is tightly linked to the generation of highly immunogenic antigens. This provides a basis for immune-modulatory therapies, e.g., using immune checkpoint inhibitors, which can reactivate a pre-existing, but exhausted immune response. However, for a largely applicable preventive approach, the predictability of the antigens plays a crucial role. The Darwinian selection principles behind the evolution of MSI cancers allow the prediction of antigens before a cancer develops (**Figure 1A**). As microsatellite mutations that confer proliferation and growth advantage (by disabling tumor suppressor genes while evading immune recognition and elimination) will be selected for, the respective antigens are over-represented in cancer precursors and manifest cancers and thus serve as promising vaccine targets.

Using a comprehensive bioinformatics approach, we and others previously characterized cMS across the human genome, establishing two major findings: (1) the mutation frequency of a microsatellite largely depends on its length, following a sigmoid curve; (2) cMS mutations with a frequency higher than what was predicted based on microsatellite length are the likely drivers of tumorigenesis, reflecting selection during MSI cancer evolution. Using the information about length-adjusted mutation frequency, it is possible to predict the relevance of specific mutations in the carcinogenic process and their frequencies.

Using this approach, we were able to trace the evolution of MSI cancers down to a few recurrent mutations shared across tumors and patients, opening a new avenue in the field of cancer prevention. By identifying key driver mutations in the MSI carcinogenic process, we predicted the resulting peptide structures resulting from these mutations and demonstrated their ability to induce T cell responses *in vitro* (47, 84) (**Figure 1B**). In the next step, these candidates were combined in a trivalent vaccine containing three recurrent and immunogenic antigens derived from AIM2 (-1 deletion), TAF1B (-1 deletion) and HT001 (-1 deletion) frameshift cMS mutations that were shared by more than 85% of MSI tumors. This vaccine was evaluated in a first-in-human Phase I/IIa clinical trial analyzing the safety and immunogenicity of a cancer vaccine in a total of 22 patients (48). The study demonstrated a favorable safety profile with no treatment-related severe systemic adverse effects observed in any of the vaccinated patients. However, grade 2 local injection site reactions have been observed in 3/22 vaccinated study participants, indicating that vaccination-related side effects need to be accounted for in future vaccine formulations and strategies, particularly in LS carriers with pre-existing FSP-specific immune responses. Importantly, all patients vaccinated per protocol demonstrated FSP-specific cellular (predominantly CD4 T cells) and humoral immune response against at least one vaccine antigen.

Although the results of the first clinical trial with the trivalent FSP peptide vaccine described above were highly encouraging, antitumor efficacy was not the primary objective, which would have required a long-term follow-up if tumor recurrence was used as the primary endpoint. To ask whether the FSP-based cancer vaccine could prevent or intercept CRC tumorigenesis, pre-clinical studies in mouse models have been performed. Based on the *VCMsh2* LS mouse model developed by Kucherlapati and Edelman et al. (85), which recapitulates human LS-associated intestinal tumorigenesis by biallelic Villin-dependent conditional knockout of *Msh2* in the entire intestinal epithelium, the preventive effect of rFSP vaccination was evaluated. Bioinformatics analysis of 488,235 cMS in the murine genome combined with the gene expression and mutation frequency data identified thirteen candidates possibly relevant for the MSI tumorigenesis in Lynch mice (86). The immunological assessment including epitope prediction and immunogenicity analysis revealed four promising candidates for vaccination. Vaccination with these candidates, Nacad (-1 deletion), Maz (-1 deletion), Senp6 (-1 deletion), and Xirp1 (-1 deletion) alone or in combination with non-steroidal anti-inflammatory drugs (NSAIDs) (aspirin or naproxen), which have been examined for chemo preventive effects in LS patients (9, 87, 88), elicited robust T cell immune responses as measured by IFNγ ELISpot and a significant tumor-preventive effect in *VCMsh2* mice. Interestingly, the tumor-preventive effect was strongest in the rFSP vaccine plus naproxen combination arm, supporting the hypothesis that NSAIDs may enhance vaccine-induced antitumor efficacy by reshaping the immune microenvironment in the intestinal mucosa and enhancing immune surveillance (89, 90). Clinically, this suggests that the reduction of tumor incidence by NSAIDs, reported for Lynch carriers in retrospective (91) and controlled prospective studies (9, 87, 88), could further be enhanced by FSP vaccines.

In addition to the peptide based FSP vaccination approach, further studies pursuing a different vaccination approach are currently underway, including viral vector-based FSP antigen delivery with a substantially higher number of antigens (over 200), which have been derived from human MSI tumors as analyzed by *in vitro, in vivo* and *ex vivo* tools. The study is currently recruiting LS patients for evaluation in clinical trials (92).

## HLA genotype and tumor antigen evolution

Both clinical and preclinical data on FSP vaccines’ effectiveness hinted at the importance of epitope selection for eliciting CD4 or CD8 T cell responses. Although the dominant role of cytotoxic CD8 T cell response has been suggested in the efficacy of tumor cell elimination, immune responses engaging CD4 T cell response have been gaining more relevance with growing knowledge in tumor immunology (93, 94). Specifically, the human trivalent FSP vaccine trial (48) demonstrated that FSP-specific immune responses were predominantly mediated by CD4-positive T cells, whereas less than 50% of vaccinated patients developed significant CD8-positive T cell responses. Although this may in part be related to the use of long peptides and Montanide ISA51 used as an adjuvant, the response pattern may also reflect the availability of epitopes in the FSP sequences compatible with the HLA genotype of vaccinated individuals.

The induction of cellular immune responses requires the presentation of epitopes through HLA (or MHC) molecules. Whereas CD4-positive T cell receptors interact with HLA class II molecules, CD8-positive T cells interact with HLA class I molecules. As the structure of the HLA molecules determines the binding affinity and presentation of certain epitopes (95, 96), the HLA type of an individual influences the epitope repertoire presentable by tumor cells and antigen-presenting cells. As the repertoire of HLA molecules is large and the HLA class I and HLA class II antigen-encoding gene loci are highly diverse, the clinical efficacy of cancer vaccines will vary depending on an individual’s HLA genotype. Observations from the recent COVID-19 pandemic regarding the disease course and responses to vaccination with SARS-COV-2 antigens support this association (97–99). Thus, although the shared nature of the driver cMS mutations among different patients and tumors allows the use of a limited set of recurrent candidate neoantigens, response to vaccination and tumor-preventive effectiveness might be substantially improved by the adaptation of the epitopes derived from these neoantigens to the specific HLA type of an individual. Accounting for HLA type specificity is therefore an important task for future immune prevention and interception approaches, potentially enabling higher vaccine effectiveness by individualization of the vaccine formulation to a person’s HLA genotype (100).

It is plausible to assume, that HLA genotype influences not only immune responses induced by vaccination, but also natural immune responses in LS carriers prior to and after tumor manifestation (47, 101, 102). If HLA type in fact influences immune surveillance, the HLA genotype may influence the tumor incidence or tumor risk in LS carriers. Studies analyzing the possible effect of the individual HLA type on cancer risk in LS have been initiated (100, 103). The findings expected from these studies will guide the future development of next-generation HLA-adapted individualized neoantigen vaccines.

Significant recent advances in vaccine technology and immunology indicate that individualized vaccine strategies hold potential for future cancer immune prevention and interception. At the same time, this strategy poses a challenge to the production process of vaccines. Even if certain HLA genotypes with similar peptide-binding characteristics can be united under an HLA supertype, vaccine formulations need to be adapted and produced on a relatively short-term. Peptide-based vaccination, though offering a robust and well-studied and evaluated technology, may lack sufficient flexibility for adaptations, particularly in the scenario of therapeutic application in patients with manifest cancer.

**Figure 2:**
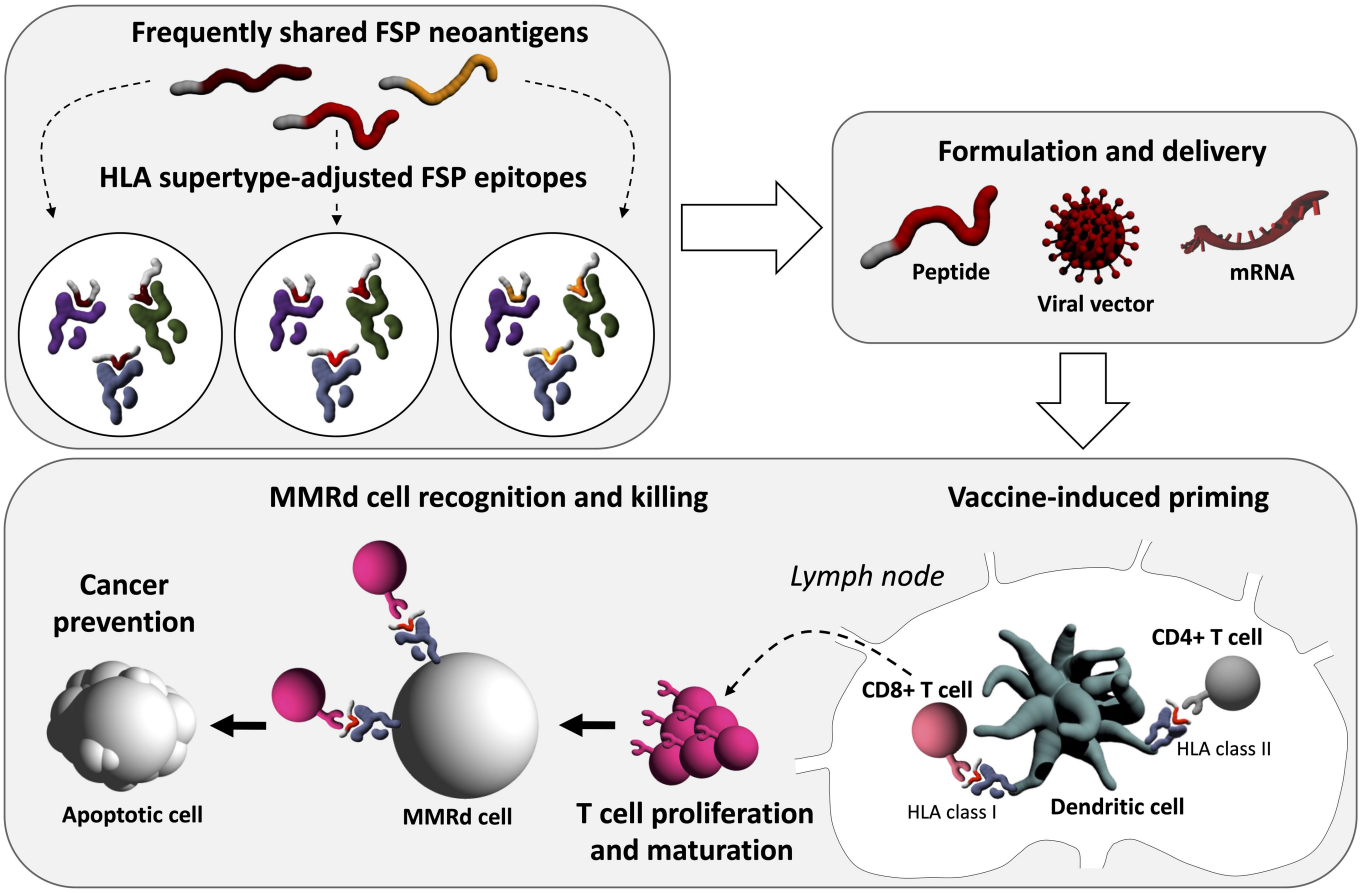
Conceptual illustration of next-generation FSP preventive cancer vaccines. After the selection of immunogenic FSP frequently shared among MMRd cells, FSP epitopes can be HLA supertype-adjusted, facilitating higher vaccine effectiveness. Vaccine delivery can be pursued, among others, with different approaches including peptide, viral vector-based, or mRNA technology. Immunologically, the first step will be vaccine-induced priming, during which the delivered neoantigens encounter antigen-presenting cells, most importantly dendritic cells at the injection site. Neoantigen-loaded dendritic cells traffic to lymph nodes which are primary sites of T cell priming. There, vaccine-derived antigens on HLA class I and II molecules are presented to CD8^+^ and CD4^+^ T cells. Activated T cells proliferate and mature into effector T cells which leave the lymph nodes entering the periphery. Ideally, neoantigen-specific CD8^+^ T cells can induce apoptosis in MMRd cells, which present the respective antigen on HLA class I molecules, and thereby prevent cancer.

The application scenario (preventive, interceptive or therapeutic) also directly affects the maximal possible level of individualization. Preventive applications can maximally target likely antigens derived from predictable mutation events. The selection of candidate epitopes is restricted to the candidate antigens proven highly relevant and immunogenic. Personalization according to the current knowledge would concern the HLA genotype-adjusted epitope selection, although not the candidate spectrum itself. Such “warehouse” vaccines (104, 105) transferred to the high-risk scenario of LS would focus on the most frequently shared FSP neoantigens adjusted to the predominant HLA alleles (**Figure 2**). In this setting, peptide-based vaccine may possibly offer a time- and cost-saving solution. In a therapeutic or intercepting approach, the presence of a manifest tumor would enable the analysis of tumor mutanomes and peptidomes (36), binding affinity to the specific HLA molecules of the patient, and immunogenicity, thus enabling the construction of a vaccine based on the given specifics of the tumor and patient. However, this process is time-consuming and may not be ideal for timely initiation of interventions required for cancer interception and treatment.

Throughout the continuum of tumor development, the immune system constantly interacts with emerging tumor cell clones, the process known as tumor immunoediting. When developing immunopreventive or immunotherapeutic approaches, tumor immune evasion should be taken into consideration, as the host immune system will not recognize “escape” tumor clones effectively. The interplay between arising tumor cell clones and host’s immune cells and characteristics of tumor evolution have been illustrated by several studies (101, 106–110). Such an interplay is particularly pronounced in the scenario of the highly immunogenic MSI cancers. Among those, the ones with LS background could be exposed to a longer process of immunoediting, as the LS carriers have been reported to present with microscopic lesions with normal histomorphology but lacking MMR protein expression (111). Such lesions were proposed to induce the systemic and local immune responses measurable in the blood and colon of tumor-free LS carriers, respectively (47, 102), suggesting the process initiated long before a clinically detectable tumor. We have previously shown that higher frameshift mutation frequency is correlated with lower immunogenicity of resulting neoantigens, whereas a lower frequency of mutations correlated with highly immunogenic antigens (101). This inverse correlation suggests counter-selection of cell clones expressing highly immunogenic neoantigens according to the tumor immunoediting concept. On the other hand, such counterselection may be negated in cases where antigen presentation machinery is dysregulated. In fact, general alterations of the antigen presentation machinery are common in Lynch-associated cancers and observed at higher frequency in advanced lesions. Such evasion phenomena, including complete breakdown of HLA class I-mediated antigen presentation following mutations of the Beta-2-microglobulin (B2M) gene, can interfere with the effectiveness of vaccines. Immune evasion is considered one of the most important reasons for the limited success of previous cancer vaccine trials with a therapeutic design. Therefore, transferring vaccine approaches towards earlier stages (interception) or entirely to cancer prevention in high-risk individuals marks a paradigm change with high potential for re-shaping the field of anti-cancer vaccines and cancer prevention in general. The complexity of this biological process and limited possibilities for experimental investigation calls for mathematical modeling approaches that could account for possible variables and predict the repertoires of relevant antigens or even model the possible outcomes of immune interception approaches.

Advances in next-generation sequencing together with the development of *in silico* epitope prediction algorithms and *ex vivo* assessments of immune responses revolutionized the identification of tumor neoepitopes suitable for cancer vaccines (30, 112). Tumor neoantigens originating from genomic alterations beyond those resulting from MMRd-triggered frameshift mutations can also be targeted by the immune system as non-self, leading to tumor-specific T cell responses (32, 113, 114). In fact, vaccination strategies targeting specific tumor neoantigens have demonstrated effective T cell responses against tumor specific antigens and potential clinical benefit (41, 42, 115–117). Neoantigen-based cancer therapies are highly personalized, requiring the development of a vaccine for each individual patient, which limits scalability and availability. To ensure broader applicability, cancer vaccines targeting shared immunogenic neoantigens originating from functionally relevant driver mutations on genes such as KRAS (114, 118), TP53 (119, 120), BRAF (121), PIK3CA (122), and EGFR (123, 124) or from recurrent gene fusions typically occurring in sarcomas (125, 126) have been pursued. However, compared to FSP in LS cancers, the pool of such antigens is limited and their effectiveness in an HLA-diverse population needs to be investigated for other HCS setting. Therefore, the development of personalized cancer vaccines may be warranted for cancer prevention in other HCS carriers, who unlike in LS do not share recurrent neoantigens for “off-the-shelf” vaccine formulations with defined and validated neoantigens. Neoantigen based approaches are promising and likely to improve further with advances in neoantigen prediction pipelines.

**Figure 3:**
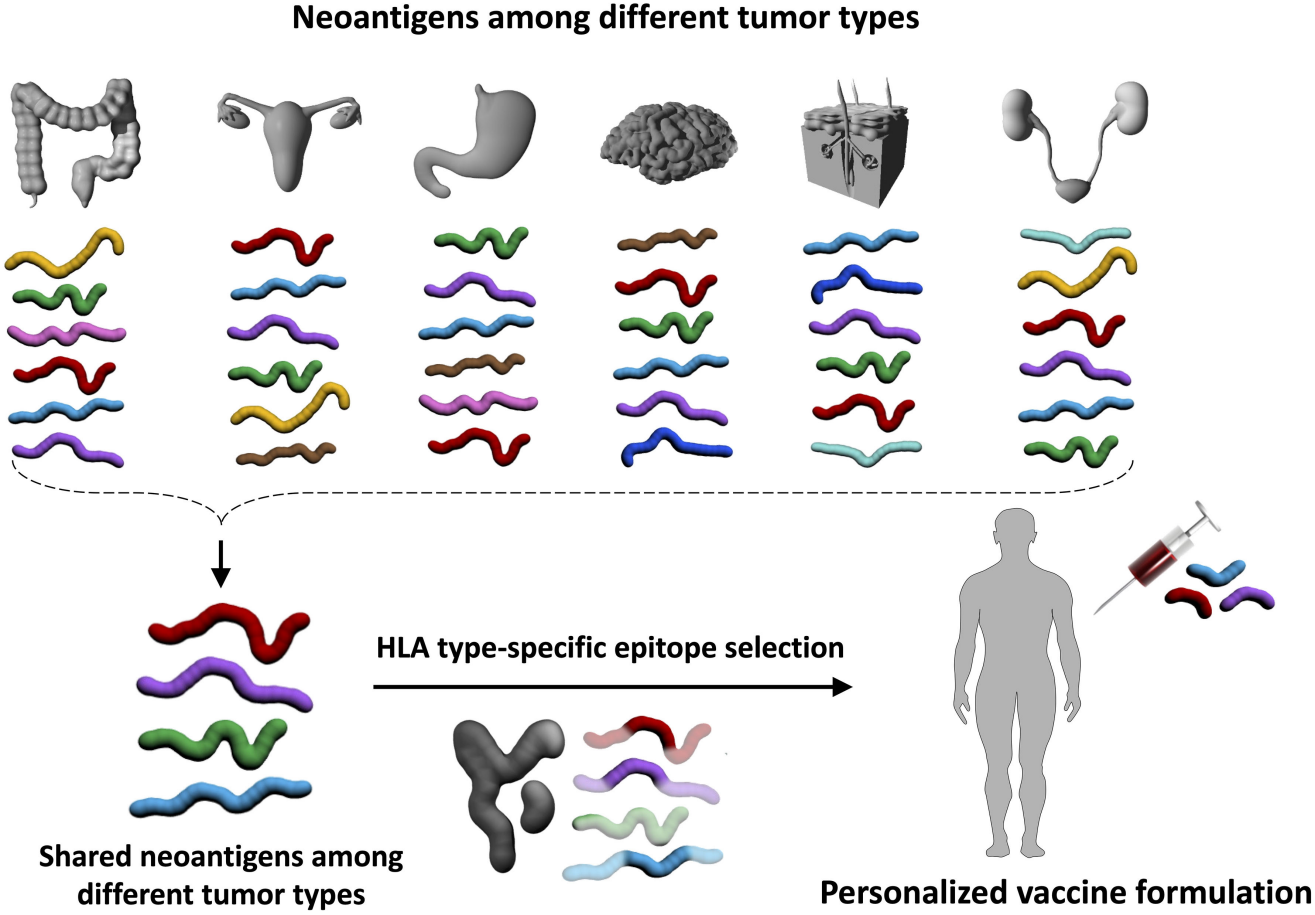
Immunopreventive potential of neoantigens shared by tumors in different organs. In addition to the characteristic LS-associated tumors in the colorectum and endometrium, the tumor spectrum in LS also encompasses other organs such as brain, skin, and kidney tumors. It is possible that certain neoantigens are shared across different tumor types (red, purple, green, and aqua blue antigens in the upper panel). Immunopreventive vaccines with these shared neoantigens that are further adjusted by an individual’s HLA genotype may allow the development of a personalized, preventive vaccine without organ restriction. Such an approach would be particularly valuable for cancers without screening options. This is illustrated by the example in the lower panel of the figure, where one of the shared neoantigens (green) is not included in the vaccine formulation because this green antigen does not contain neoepitopes that bind to the individual’s HLA molecules.

Computational tools predictive of peptide binding affinity to specific HLA molecules are available (96, 127–129). These are typically neural network-based algorithms that were trained using existing allele-specific peptides such as those stored in the Immune Epitope Database (130) and Dana-Farber Repository for Machine Learning in Immunology (131). Recently, HLA immune-peptidomics-based approaches to discover HLA-restricted peptides generated large-scale datasets of endogenous HLA-bound peptides that resulted in the development of more accurate epitope prediction algorithms. These algorithms not only predict epitope binding to a specific HLA allele but also consider epitope expression and proteasomal processing to predict epitope presentation more accurately (132). The final frontier in the prediction of neoantigens is the development of an algorithm that can accurately predict antigenicity. Neoantigens can be presented on surface HLA, however not all presented epitopes generate a T cell response, and even presented viral epitopes may not be recognized by T cells (41, 42, 115–117, 133) in some cases. The development of an accurate machine learning algorithm requires thousands of validated T cell epitopes per HLA allele, which are currently unavailable for many alleles. There are ways to overcome this obstacle. Researchers observed more stable HLA ligands yield more immunogenic epitopes (134, 135) and epitopes mimicking pathogen-derived known antigens are more immunogenic (136, 137). Incorporation of these motifs to the neoantigen prediction pipelines may improve vaccine outcomes. Utilization of all these informatics and genomics pipelines may identify validated immunogenic shared neoantigens for LS patients in an HLA-type specific manner that could be the basis of a cancer preventative vaccine. It is plausible that such vaccines may prevent not only LS-associated CRC, but also endometrial and other extracolonic tumors (61, 138) as certain neoantigens are likely shared by the spectrum of cancers arising in LS (**Figure 3**).

## Emerging technologies, research gaps, and translational barriers

Emerging new mRNA-based vaccine technologies (139, 140) have transformed the medical field by offering a technological platform with high adaptive capacity, allowing rapid translation of newly gained genomic knowledge into clinical applications for the prevention and treatment of human diseases (42, 141–143). In addition to its high immunogenicity, flexibility and versatility, relatively straight forward regulatory requirements successfully established during the COVID-19 pandemic make mRNA-based vaccination approaches attractive for personalized medical interventions such as precision cancer preventive vaccines. Other innovations in mRNA vaccine platforms include the use of self-amplifying RNA (saRNA) (144–148), circular RNA (circRNA) (149, 150) and modified LNP formulations for mRNA delivery (151–155). While these non-linear mRNA molecule-based vaccines are expected to offer “amplified” expression of encoded proteins *in vivo* requiring lower RNA doses (saRNA) and improved scalability with potentially lower toxicity concerns (circRNA), novel formulations of LNP can be developed to steer the host immune system towards mounting specific immune responses desired for the intended applications (e.g., Th1 *vs*. Th2 immunity) (156). Innovative engineering of RNA molecules and their delivery systems is expected to further help advance the optimization of next-gen RNA vaccine design strategies for precision cancer preventive vaccines for LS and other HCS.

The gold standard of screening for immunogenic recurrent neoantigens, for example for “off the shelf” vaccines, has been to test an individuals’ autologous T cells for immune responses to specific neoantigen peptides *in vitro* as measured by interferon gamma production either by ELISPOT or fluorescent activated cell sorting assays. For personalized neoantigen-based therapeutic cancer vaccines, however, because of the large number of potential candidate neoantigens, individualized immunogenicity screening is challenging and time-consuming, which is not ideal due to the urgency for starting vaccinations in patients who have established cancers. Thus, immunizing peptides were selected based on the basis of HLA binding predictions for personalized cancer therapeutic vaccines (41, 42). For off the shelf antigens, one new approach is screening in transgenic mice with human HLA. Currently, these are available for selected alleles including HLA-A2 (157), HLA-A1 (158), and HLA-B7 (159). Importantly, these models have ablated endogenous murine MHC (157). More recently, a series of HLA class I knock-in (KI) mouse strains have been generated (160). In these novel HLA class I transgenic mice, a chimeric HLA class I molecule (α1/α2 domain of HLA-A and α3 domain of H-2D^b^) was covalently linked with 15 aa to human Beta-2-Microglobulin (B2M) and introduced into the endogenous mouse *B2m* locus, resulting in the loss of endogenous mouse MHC class molecules in homozygous KI mice. HLA-restricted, epitope-specific cytotoxic T cells (CTLs) were induced in HLA KI mice upon vaccination (160). These HLA mouse models can be used as a filter towards selecting human common neoantigens as potential vaccine cargo. However, given the diversity of human HLA alleles (161), and the lack of HLA-C mouse models, these models can only be used for frequent HLA alleles.

As with any other agents under development, the demonstration of efficacy is of paramount importance to the successful development of cancer preventive vaccines. If a cancer-free period is used as a primary endpoint for efficacy in cancer prevention studies, however, it will require a larger number of study subjects and long-term follow-up in order to obtain conclusive evidence (162, 163) even in the LS and other cohorts with an increased cancer risk. Therefore, potential surrogate biomarkers, if carefully selected and included in cancer prevention clinical trials, will help delineate clinical correlates of cancer-preventive efficacies. Long-term fortification of the host immune defense against cancer is the ultimate goal of cancer preventive vaccines. Such vaccines must be able to drive and maintain antitumor immune surveillance that can effectively intercept and eliminate emerging tumor precursor cells while avoiding the immune evasion. Because immune biomarkers of cancer preventive vaccines’ efficacies have yet to be fully elucidated, multi-pronged research strategies are needed to establish immune correlates of protection, including emerging knowledge from immune biomarker studies conducted in cancer patients (164, 165), which may inform the direction of research. For example, clinically beneficial adaptive antitumor immune responses have been characterized locally in the TME and systemically. Tumor infiltrates in the TME with higher densities of antigen-specific Th1 cells, CTLs, and memory T cells, and lower densities of immunosuppressive T regulatory cells and myeloid derived suppressive cells (MDSC) are generally predictive of better outcomes in cancer patients (164, 166–172). Systemically, immune signature of more favorable responses to ICI immunotherapy has been observed in patients, who at baseline had a diverse TCR repertoire (173), a higher number of CD8^+^ effector T cells in the periphery and at the tumor margin (174, 175), and a lower level of MDSC (176–178), and had higher levels of TCR repertoire (173, 179), increased levels of CD127^low^ PD-1^low^ CD4 T cells (180), and peripheral expansion of CD8^+^ T cells (181–183) at post-treatment. Evaluation of some of these immune biomarkers that are linked to favorable clinical outcomes should be included in preclinical and clinical studies of candidate cancer vaccines, so the immune response profiles can be correlated with *in vivo* antitumor efficacies observed in vaccinated animals and with surrogate biomarkers of efficacies in human study subjects, respectively.

More recently, the roles of tissue-resident memory CD8^+^ T (T_RM_) cells have been extensively studied in cancer immunosurveillance. T_RM_ cells are known to function as “pathogen alert” system against invading pathogens for the local organ systems (184–187). Mounting evidence suggests that T_RM_ cells are a critical component of the host immune surveillance and defense mechanisms against developing cancer (184–186, 188–190). A higher number of intratumoral T_RM_ cells is predictive of better overall survival (191–194). Mechanistically, these cells in the TME express immune checkpoint receptors (ICR) and exert antitumor effector functions when ICR are blocked by ICI (188, 189, 195), thus linking the presence of T_RM_ in the TME to more favorable responses to ICI in cancer patients. Furthermore, T_RM_ cells have been shown to recognize neoantigens (192, 193, 196) and can amplify ICI-mediated antitumor immunity not only by exerting effector functions but also promoting epitope spreading through dendritic cells (193, 196). Preclinical studies have shown T_RM_ cells can be induced by vaccination against tumor neoantigens and that vaccine-induced T_RM_ cells potentiated the host antitumor immunity, rejecting tumor challenge (192, 197, 198). In contrast to clinically beneficial cell-mediated antitumor immune responses widely reported to date, the prognostic value of B cell-mediated humoral immune responses in cancer patients has yet to be fully elucidated (164). Recently, a higher number of B cells in the TME and the presence of intratumoral tertiary lymphoid structures, which include B cell follicles as well as T cells, macrophages, and dendritic cells, have been observed in patients who had better clinical outcomes (164, 199). The role of B-cell mediated immunity for cancer control warrants further investigation.

As discussed earlier, in the first clinical study with trivalent FSP vaccine in patients with LS-associated or non-LS associated MSI-H CRC, all evaluable vaccinated patients showed FSP-specific humoral and predominantly CD4^+^ T cell responses (48). In the *VcMsh2* LS mouse study discussed earlier, murine rFSP vaccination elicited robust FSP antigen-specific T cell responses (CD4^+^ and/or CD8^+^) and humoral immune responses systemically and upregulated intratumoral Th1 signaling pathway more so than Th2. Moreover, intestinal tumors from vaccinated *VcMsh2* mice had significantly elevated levels of CD4^+^ and CD8^+^ T cell infiltrates as compared to control tumors (86). These immune response findings are consistent with what has been reported clinically and suggest that rFSP vaccine-induced immune responses were responsible for the observed cancer preventive efficacy *in vivo*. Cancer preventive vaccines, regardless of whether based on commonly shared tumor antigens or personalized neoantigen repertoire (predicted or omics-informed), should be able to at a minimum elicit clinically beneficial antitumor immunity discussed above ideally with long-term memory. To learn and establish immune correlates of protection against cancer, these immune parameters should be included in clinical trials for cancer preventive vaccines as part of investigative biomarker analysis.

In the premalignant and pre-invasive stage setting, there is accumulating evidence to suggest pro-tumorigenic MDSC and immune escape mechanisms mediated through immune checkpoints and immune-suppressive interleukins are already present and contributing to the malignant progression (200–202), which may suppress or hinder adaptive antitumor immune responses to cancer vaccines. Immune profiling of LS polyps has previously demonstrated a significantly increased level of pro-inflammatory and immune checkpoint molecules (203). Cancer vaccine-induced antitumor immunity, therefore, may not be sufficient to effectively prevent or arrest tumorigenic process especially in the setting of intercepting premalignant lesions. According to current knowledge, about two-thirds of LS CRC develop from MMRd crypts, suggesting loss of function of the MMR system as the initiating somatic event (204–206). In such a scenario, the generation of MMRd-triggered FSP likely precedes local immunosuppression, potentially opening a window for vaccine-mediated interception. However, the differential effectiveness of rFSP vaccines for the prevention of LS CRC triggered by MMRd *vs*. those resulting from MMR-proficient adenomas and subsequent MMR inactivation needs to be evaluated in future studies.

The addition of immunomodulatory agents to cancer vaccines may be warranted to induce more robust adaptive immune responses for LS and other high-risk cohorts. For example, chemo preventive effects of NSAID (aspirin and naproxen) have been extensively studied in LS patients (9, 87, 88). In addition to directly reducing the level of pro-tumorigenic prostaglandin E2 (88), naproxen has been shown to potentiate antitumor immune responses by rFSP vaccination in the *VcMsh2* mouse study (86) and boosted immune surveillance in LS patients (88). In preclinical models of CRC tumorigenesis, naproxen administration has been shown to decrease the expression of PD-L1 in colon tumors and increase the density of CD8^+^ TILs (207). Since the efficacy of ICI has already been demonstrated in LS and MSI-high cancer patients, ICI-based treatment is being considered for immunointerception in the premalignant setting in LS cohort (208). There are other classes of immunomodulatory agents that can be potentially used to boost the host immune responses to cancer-preventive and interceptive vaccines (209–212). Feasibility, efficacy, and safety of combination of cancer preventive vaccines and these newer immunomodulatory agents should be explored especially for LS and high-risk cohorts through preclinical and clinical research.

## Concluding remarks

The first clinical study with FSP neoantigen-based cancer vaccine (NCT01461148) was launched more than a decade ago in MMR-deficient colon cancer patients (48). This seminal study, which was built on the culmination of many years of extensive research on LS tumor molecular biology and endogenous immunity led by the same group, Kloor, von Knebel Doeberitz, and their colleagues, has dramatically changed the landscape of neoantigen-based cancer vaccine research. Over the last decade, there has been an explosion of research on tumorigenesis and genetic triggers, tumor immune surveillance, immune checkpoint mechanisms that can unleash antitumor immunity, contexture of tumor-immune microenvironment, and dynamic interplay between evolving tumors and immune defense, all of which are generating the consensus that better cancer control and favorable outcomes are achievable if tumorigenesis is intercepted earlier than later. Together with technological advances in tumor genomic landscape profiling, cancer vaccinology, and innovative immunomodulatory agents, precision cancer prevention and interception for LS carriers is within the reach. There are, however, remaining questions that must be addressed. For example, even if technical challenges of personalized FSP vaccine production can be overcome, can personalized neoantigen-based precision cancer vaccines lead to more efficacious and long-term immune protection than shared FSP neoantigen-based vaccines in LS carriers? To remain cancer free, how long do LS carriers need to maintain antitumor immune memory? Does the combination of immunomodulatory agents help sustain the durability of immune protection in LS carriers? There are translational barriers that also need to be overcome before the true benefit of precision cancer preventive vaccines are realized for LS carriers. The preclinical research field will greatly benefit from better preclinical models that can more closely mimic human LS tumorigenesis and human immune system. Newer generation of humanized preclinical models may help bridge the inter-species knowledge gap that has been a major obstacle in translational research for LS and other cancer vaccines. Lastly, as next-generation novel surrogate markers emerge from preclinical and clinical studies in the next decade, regulatory approval pathways will have to be reviewed and improved for scientific harmonization without delay. The success of FSP neoantigen-based cancer vaccines for LS cancer prevention will hopefully demonstrate the potential marketability of cancer preventive vaccines in the next decade, which will bring an increasing interest from the private sector and can lead to the partnership opportunities between academia, government, and industry for the betterment of quality of life for LS and other high-risk populations.

## Author contributions

SS, AA, DK, SL, and MK wrote this review. LB, JG, and MKD critically reviewed and revised the manuscript. All authors contributed to the article and approved the submitted version.
